# The complete chloroplast genome of *Pterospermum menglunense* (Sterculiaceae), an endangered species

**DOI:** 10.1080/23802359.2021.1907251

**Published:** 2021-03-26

**Authors:** Yang Guan-Song, Lei Peng, Zheng-An Yang, Jie Zhang, Ying-An Zhu, Jun-Jun Xie

**Affiliations:** College of Horticulture and Landscape, Yunnan Agricultural University, Kunming, China

**Keywords:** *Pterospermum menglunense*, endangered species, complete chloroplast genome

## Abstract

*Pterospermum menglunense* is the endangered plant species of the genus *Pterospermum* in the family Sterculiaceae. In the study, the complete genome was 162,421bp in length, including of two inverted repeats (IRA and IRB, 25,572 bp), separated by a large single-copy region (LSC, 90,754 bp) and a small single-copy region (SSC, 20,523 bp). The genome annotation reveals a total of 132 genes, including 37 transfer RNA (tRNA) genes, 8 ribosomal RNA (rRNA) genes, and 87 protein-coding (PCG) genes. The phylogenetic tree showed *P. menglunense* is closely related to *Pterospermum kingtungense*.

*Pterospermum menglunense* H. H. Hsue is one endangered plant species of the genus *Pterospermum* in the family of Sterculiaceae (W. U. Zhengyi et al. 2005; W. Zhengyi et al. 2008). The restricted distribution range is 800–1000 m altitudes in the southern limestone mountain forests of Menglun Town, Mengna County, Yunnan Province, China (Mamun et al. 2001). This species is endemic and endangered in China. According to IUCN Local Endangered Rating Standard (Walter and Gillett 1998), it is an endangered species (EN). However, there is insufficient information about the chloroplast genes of the endangered species. In this study, the chloroplast genome of *P. menglunense* has been completed and will accordingly understand the chloroplast genome feature of Sterculiaceae (Yang et al. 2017).

The fresh leaves of a wild *P. menglunense* plant were sampled from Xishuangbanna in Yunnan Province, China (geospatial coordinates: 101.6667 E, 21.2667 N, altitude: 780 m). CTAB method was adopted to complete the extraction of total genomic DNA (Doyle 1991). The Total DNA samples (XSBN 1-3) and the Voucher specimen (*P. menglunense* XSBN 2020-11) were kept at the Molecular Laboratory, College of Horticulture, Yunnan Agricultural University, Kunming, China (Yang et al. 2017). The whole-genome fragmented a short insert 500 bp reads, and sequenced with the Illumina Hiseq 4000 (Quail et al. 2008). This approach obtained 10 G high-quality paired-end reads for the subsequent analyses. Filtered data was assembled by bowtie2 and SPAdes (k-mer parameters: -k 55, 87, 121). The GetOrganelle v1.7.0 (Jin et al. 2020) and the PGA (Qu et al. 2019) were applied to assemble and annotate the chloroplast genome, respectively, with the whole plastid genome sequences of *Craigia yunnanensis* (NC_045284.1) as reference. The complete genome was generated by Genious version 11.1.14 software (Wyman et al. 2004). The GeSeq software (Tillich et al. 2017) was used to annotate and calibrate the functional genes. To annotate the cp genome, we utilized the initial annotation of cpGAVAS (Liu et al. 2012). Annotation errors were corrected manually. The annotated chloroplast genome has been submitted to GenBank (accession number MW_421596). Raw sequencing reads used in this study was deposited in the public repository SRA with accession number SRR12744902.

The study obtained a chloroplast genome of *P. menglunense,* with the size of 162,421bp. The complete chloroplast genome has a typical quadripartite structure, and the content of CG is 36.5%. The chloroplast genome was comprising of two inverted repeats (IRA and IRB, 25,572 bp, 42.89% GC content), separated by a large single-copy region (LSC, 90,754 bp, 34.23% GC content) and a small single-copy region (SSC, 20,523 bp, 30.66% GC content), respectively. The genome annotation reveals a total of 132 genes, including 37 transfer RNA (tRNA) genes, 8 ribosomal RNA (rRNA) genes, and 87 protein-coding (PCG) genes. The 18 genes contained introns, in which 15 genes (*ndhA*, *ndhB*, *petB*, *petD*, *atpF*, *rpl16*, *rpl2*, *rps16*, *rpoC1*, *trnA-UGC*, *trnG-GCC*, *trnI-GAU*, *trnK-UUU*, *trnL-UAA*, and *trnV-UAC*) contained one intron, and three genes (*clpP*, *ycf3* and *rps12*) contained two introns.

To investigate the phylogenetic location of *P. menglunense*, the study completed a phylogenetic tree. The tree consists of 6 species: *Pterospermum menglunense* (MW_421596), *Pterospermum kingtungense* (MH_606238), *Gossypium ardoreum* (NC_016712.1), *Althaea officnalis* (NC_034701.1) (Wang et al. 2018). The genomes of *Braya humilis* (NC_035515.1) and *Betula ovalifolia* (KY_199767.1) were out-groups (Yang et al. 2019). All sequences were completed using MAFFT (version 7) and RA × ML version 8.1 with 100 bootstrap replicates (Stamatakis 2014). The phylogenetic tree displayed that *P. menglunense* is closely related to *P. kingtungense* (Figure 1).

**Figure 1.**
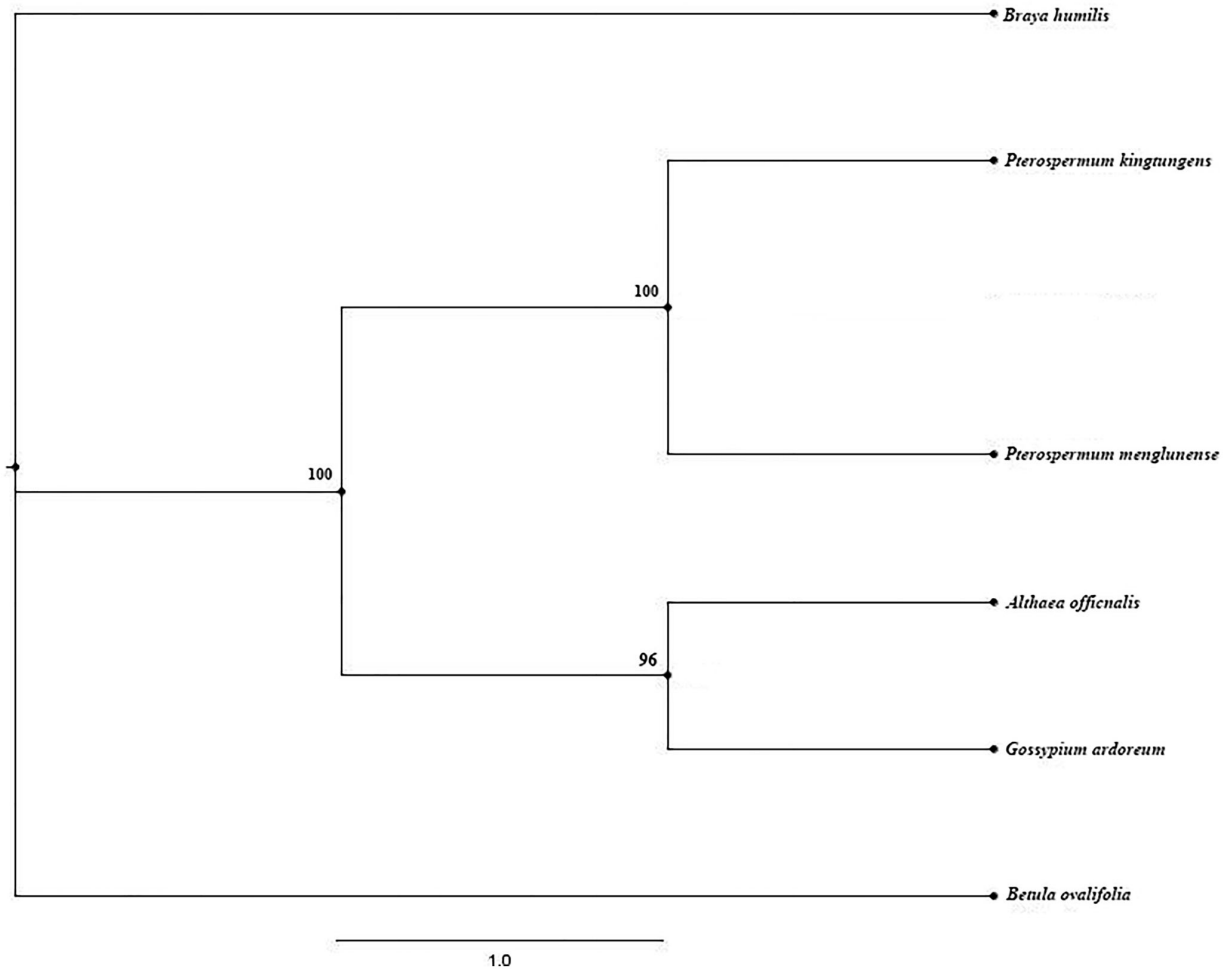
The maximum likelihood (ML) phylogenetic tree based on six complete chloroplast genome sequences. Numbers at the right of nodes are bootstrap support values.

This study provides complete information on the chloroplast genome of *P. menglunense* and contributes to its conservation and application. To determine the phylogenetic location of *P. menglunense* and *P. kingtungense*, the data would provide important information to analyze the phylogenetic relationship in the family Sterculiaceae.

## Data Availability

The data that support the findings of this study are openly available in the US National Center for Biotechnology Information (NCBI database) at (https://www.ncbi.nlm.nih.gov/nuccore/MW421596.1), reference number: MW_421596. Raw sequencing reads used in this study was deposited in the public repository SRA with accession number SRR12744902 (https://www.ncbi.nlm.nih.gov/sra/?term=SRX10312288).
